# Supplementary material for: Application of a Conducting Poly-Methionine/Gold Nanoparticles-Modified Sensor for the Electrochemical Detection of Paroxetine
Source: Polymers (Basel). 2021 Nov 17;13(22):3981. doi: 10.3390/polym13223981 (PMC8623231; doi:10.3390/polym13223981)
Supplement: Supplementary file 1 [file polymers-13-03981-s001.zip › polymers-1442742-supplementary.pdf]

List of fig caption

Figure S1: i-t curve of AuNPs electro deposition at – 0.2 V for 80 s in 1 mg/ml $HAuCl_4$.

Figure S2: Effect of step potential from 0.005 V to 0.035 V on I_{PRX} of 10 µM PRX at pH 7.5**.**

Figure S3: Effect of accumulation time $t_{.acc}$ from 2 s to 120s on I_{PRX} of 10 µM PRX at pH 7.5.

Figure S4: Effect of accumulation potential from 0 - 0.05 V on I_{PRX} of 10 µM PRX at pH 7.5.

Figure S5: EDX spectra of AuNPS GCE (I) and poly(DL-met)/AuNPS GCE.

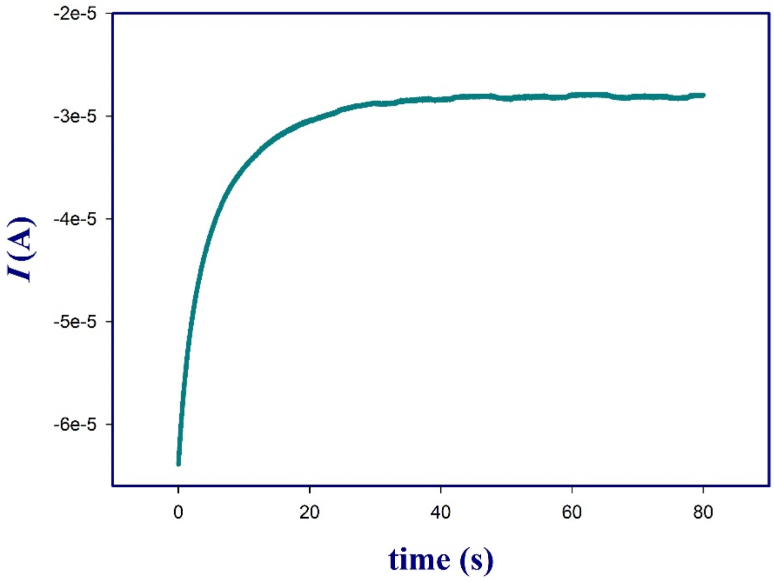

Figure S1.

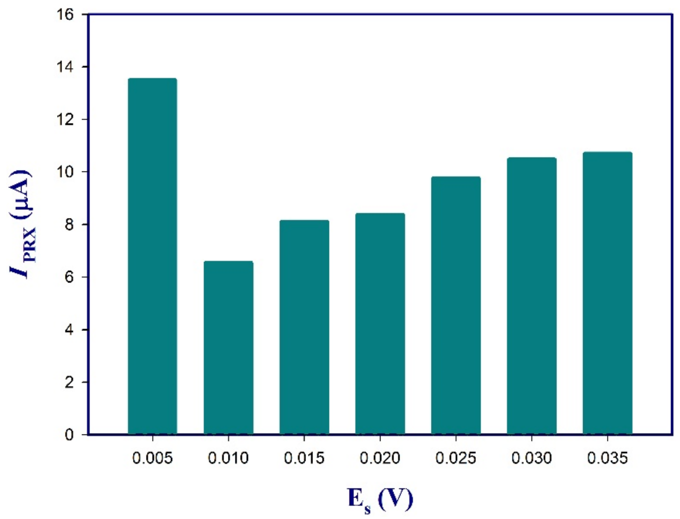

I PRX (μA)
16
14
12
10
8
6
4
2
0
0.005
0.010
0.015
0.020
0.025
0.030
0.035
Es (V)

Figure S2.

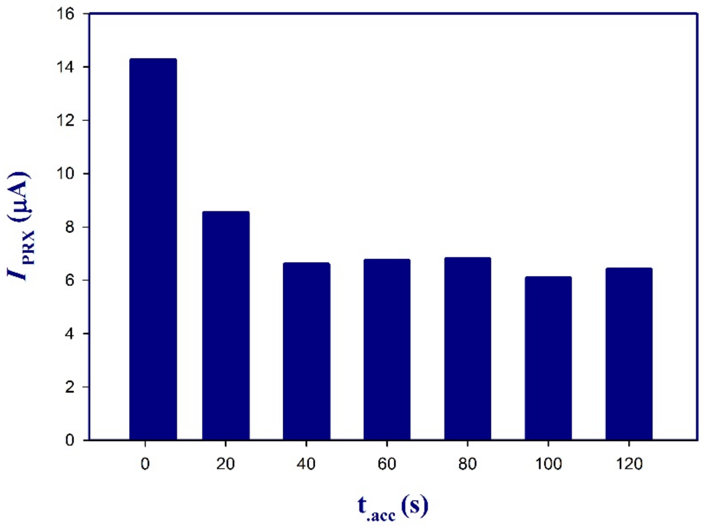

Figure S3.

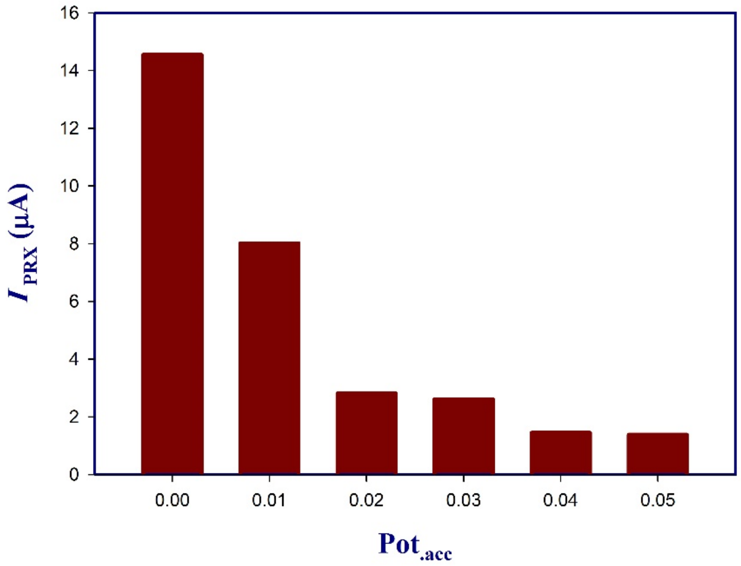

Figure S4.

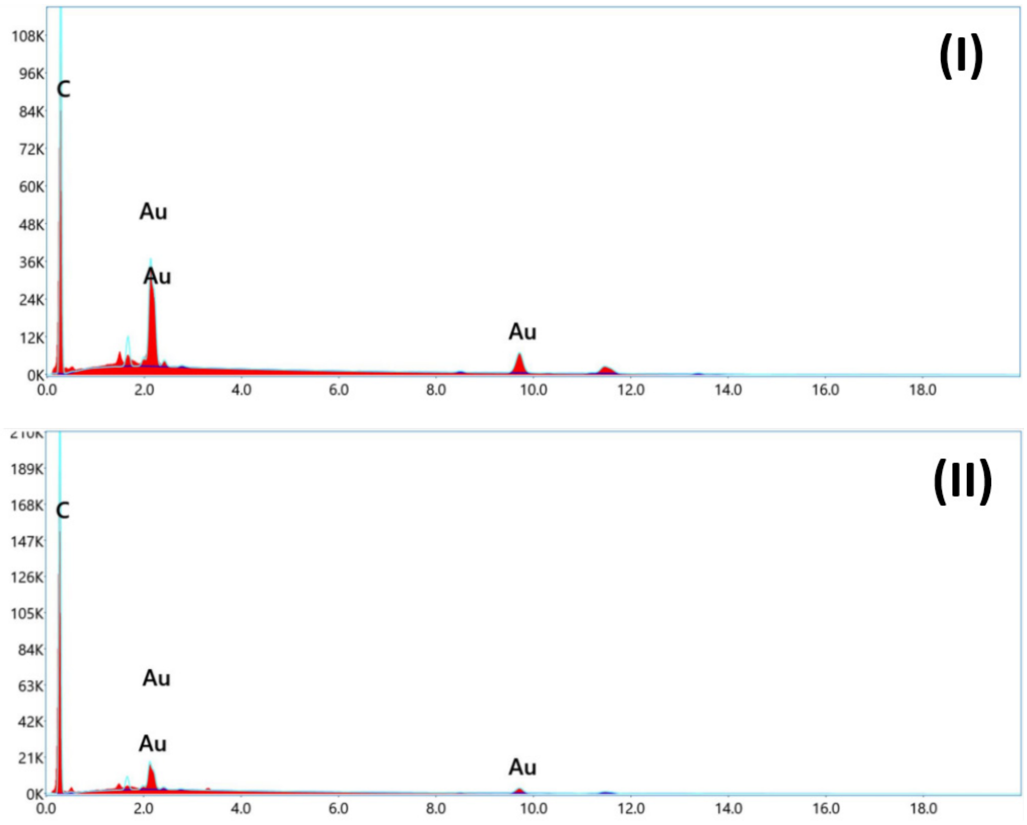

(I)
C
Au
Au
Au
108K
96K
84K
72K
60K
48K
36K
24K
12K
0K
0.0
2.0
4.0
6.0
8.0
10.0
12.0
14.0
16.0
18.0
(II)
C
Au
Au
Au
189K
168K
147K
126K
105K
84K
63K
42K
21K
0K

Figure S5.
